# Carbon Nanotube-Based Organic Thermoelectric Materials for Energy Harvesting

**DOI:** 10.3390/polym10111196

**Published:** 2018-10-26

**Authors:** Xiaodong Wang, Hong Wang, Bing Liu

**Affiliations:** 1 Frontier Institute of Science and Technology, Xi’an Jiaotong University, Xi’an 710054, China; wangxiaodongjlu@163.com (X.W.); bing.liu@xjtu.edu.cn (B.L.); 2 State Key Laboratory of Multiphase Flow in Power Engineering, Xi’an Jiaotong University, Xi’an 710054, China

**Keywords:** carbon nanotubes, composites, thermoelectrics

## Abstract

Carbon nanotubes (CNTs) have attracted much attention in developing high-performance, low-cost, flexible thermoelectric (TE) materials because of their great electrical and mechanical properties. Theory predicts that one-dimensional semiconductors have natural advantages in TE fields. During the past few decades, remarkable progress has been achieved in both theory and experiments. What is more important is that CNTs have shown desirable features for either *n*-type or *p*-type TE properties through specific strategies. Up to now, CNT‒polymer hybrids have held the record for TE performance in organic materials, which means they can potentially be used in high-performance TE applications and flexible electronic devices. In this review, we intend to focus on the intrinsic TE properties of both *n*-type and *p*-type CNTs and effective TE enhanced strategies. Furthermore, the current trends for developing CNT-based and CNT‒polymer-based high TE performance organic materials are discussed, followed by an overview of the relevant electronic structure‒TE property relationship. Finally, models for evaluating the TE properties are provided and a few representative samples of CNT‒polymer composites with high TE performance are highlighted.

## 1. Introduction

Renewable green energy has gained growing attention since traditional fossil fuels are not renewable and their widespread use is causing more and more environmental issues [1,2,3,4]. Thermoelectrics (TEs) is considered an important supplement to renewable green energies such as solar, wind and nuclear energy, etc. [5,6]. This technology offers a promising way to convert heat directly into electricity without releasing any pollution [7,8]. TE devices are generally comprised of all solid components without any moving parts or fluids, which, therefore, can work alone quietly for a very long time without maintenance and require only small temperature gradients [9,10]. Therefore, they can be combined with numerous heat sources, including abundant solar, geothermal and waste heat from automobile or industrial factories and even low-density heat from the human body, to generate clean electricity [11,12,13,14]. However, the application of TE devices is dictated by the cost and heat-to-electricity conversion efficiency of TE materials [15]. The properties of TE materials cannot fulfill the requirements for wide commercial applications right now. Exploring cost-effective materials with high TE performance (figure of merit, *ZT*) is of great interest to researchers.

Significant progress has been achieved in the TE field in recent decades. State-of-the-art inorganic materials have high *ZT* values over 2 [16,17,18,19]. However, these inorganic materials typically are expensive, heavy, brittle and contain toxic elements such as Bi and Sb [20,21,22]. A lot of researchers have turned to organic materials for low-cost, high-performance TE materials because organic materials, especially polymers, are normally cheap, low-weight, flexible and non-toxic [23,24,25,26]. In addition, solution processing and printing techniques can be used to make the manufacture of organic TE modules much easier compared to inorganic TE materials [27,28,29,30,31]. In this regard, organic TE materials have the potential to bring about significant improvement even if their conversion efficiency is comparable to, or less than, that of the state-of-the-art inorganic materials [32,33,34]. Most of the power generation by TE materials is still suitable for organic materials, although the working temperature of organic TE materials may be lower than that of inorganic TE materials due to the decomposition of organic materials at high temperature [35,36]. For example, a major part of waste heat lost from fossil fuels is released under 150 °C, as reported by Shindo et al. [37].

**Figure 1:**
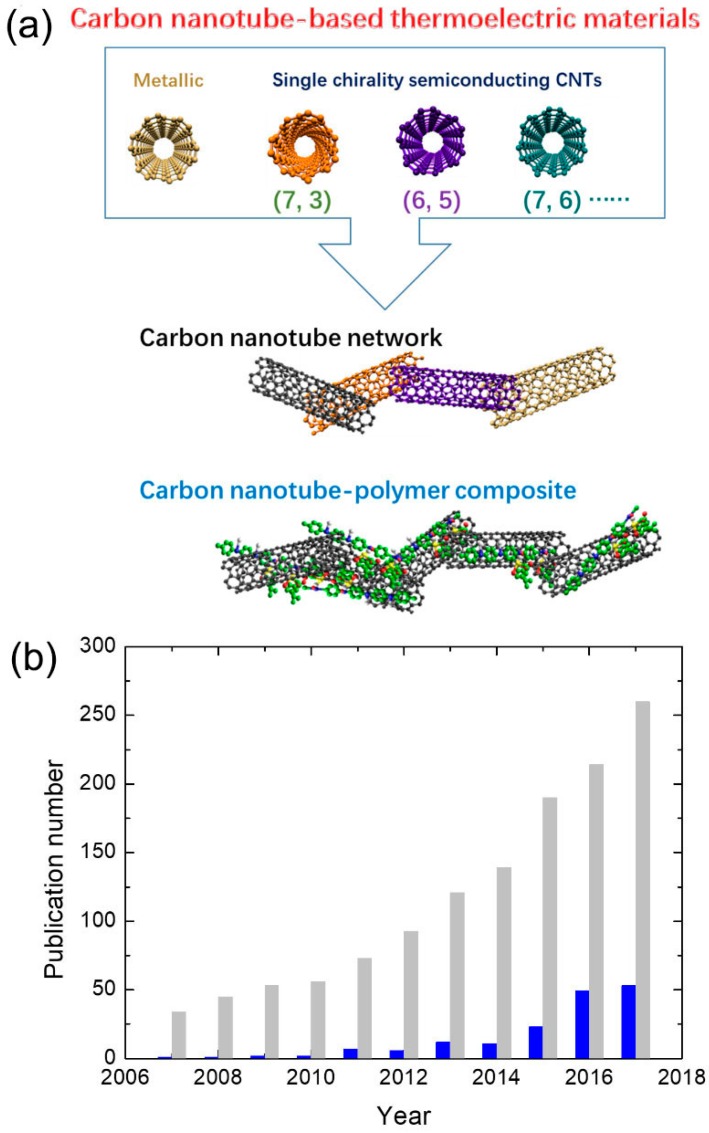
(**a**) Illustration of carbon nanotube-based thermoelectric materials: carbon nanotube network and carbon nanotube‒polymer composite. (**b**) Progress of publication number on organic TE materials and carbon nanotube (CNT)-based organic TE materials in a year according to Web of Science on 18 August 2018. Gray column: “thermoelectric” + “organic”. Blue column: “thermoelectric” + “organic” + “CNT”.

Organic materials are of great interest to researchers pursuing low-cost, flexible and high TE performance materials. After about 10 years of development, a record TE performance of organic materials has reached up to *ZT* = ~0.5 at room temperature, which is comparable to that of many inorganic materials [38,39,40,41]. The growth in number of publications per year about organic TE materials has been exponential in recent years (shown in Figure 1), which indicates growing interest in developing high TE performance organic materials in science and technology. Among these organic TE materials, CNT-based organic TE materials (shown in Figure 1a) play an important role. Making CNT hybrids is one of the most widely used strategies to design high TE performance organic materials [42]. Right now, CNT hybrids -hold the record for TE performance in organic materials [38]. The number of publications per year about CNT-based organic TE materials is also increasing fast, reaching about 53 papers in 2017 (shown in Figure 1b), which accounts for one-fifth of organic TE materials-related papers.

In this review, strategies for designing CNT-based high TE performance organic materials (CNT only and CNT hybrids) will be described after a brief introduction to the advantages of CNTs in thermoelectrics and an account of the history of and recent progress in both theory and experiments. We would also like to recommend some good review papers to readers. A good introduction to the solid-state physics of conjugated polymer-based TE materials was by Bubnova and Katz et al. [43,44]. The relationship between the chemical structure of organic materials and their TE performance has been discussed by Kroon et al. [45]. The TE properties of the most studied polymers and small molecules with charts of TE parameters have been summarized by Zhang et al. [46]. The progress in CNT-based TE materials has been reviewed previously by Blackburn et al. [47], emphasizing the fundamental TE properties of single-walled CNTs, nanotube-based composites, and TE devices prepared from these materials. Different from previous review papers, we would like to focus our attention on the intrinsic properties of CNTs in theory and experiments, and we intend to summarize and discuss the strategies that were used to improve the TE performance of CNT-based organic materials, such as tuning the carrier concentration, creating energy barriers, and making non-percolated nanostructures.

## 2. Basic Description of the Three Key Parameters for TE Materials

TE materials can convert heat to electricity through the Seebeck effect, in which carriers move from the hot side (high entropy) to the cold side (low entropy) [48,49]. The conversion efficiency is determined by three key parameters: the Seebeck coefficient (thermopower, S), electrical conductivity (σ), and thermal conductivity (k).

The Seebeck coefficient is the most important parameter among the three because the square term in Equation (1) enlarges the effect of the Seebeck coefficient in the dimensionless TE figure of merit:

(1) $$ZT=\frac{S^{2}\sigma T}{k},$$

where T is the absolute temperature. The Seebeck coefficient is defined as the potential (ΔV) created by the charge carriers’ movement, divided by the temperature gradient (ΔT) between the hot side and cold side, S=ΔV/ΔT. Note that the Seebeck coefficient is intimately related to the electronic structure of a material and depends on the contributions of charge carriers distribution to the conductivity at energies away from the Fermi level ($E_{F}$) [50,51]. According to the Mott formula for degenerate semiconductors, S is written as in Equation (2):

(2) $$S=\;\frac{\pi^{2}k_{B}^{2}T}{3q}\left(\frac{\mathrm{dln}\sigma \left(E\right)}{dE}\right)|E=E_{F},$$

where $k_{B}$ is the Boltzmann constant, q is the electron charge, and E is the electron energy [52,53]. For nondegenerate semiconductors, S can be written as in Equation (3) according to the Boltzmann equations:

(3) $$S=\frac{k_{B}}{q}\left[\frac{\left(E-E_{F}\right)}{k_{B}T}+A\right],$$

where *A* is the heat of transport constant. As described above, the doping of a semiconductor will determine the position of $E_{F}$ and hence the Seebeck coefficient. High doping levels will move $E_{F}$ into the conduction band and lead to the number of electronic states above and below $E_{F}$ being more equivalent, subsequently reducing S. However, doping is a commonly used method to increase the electrical conductivity of a material because electrical conductivity is determined as in Equation (4):

(4) σ=nqμ,

where n is the carrier concentration and μ is the carrier mobility [54]. The contradiction between the Seebeck coefficient and electrical conductivity is the main factor that inhibits the development of high TE performance materials. Besides looking for the peak value of power factor ($S^{2}\sigma$) via tuning the doping level, Katz et al. [44] suggested that it might be a good way to attempt to maximize the electrical conductivity (as long as the thermal conductivity does not increase much) while somehow maintaining unequal distributions in the density of states. The possible method is building nanostructures with large peaks in the density of states or making composites of materials with different carrier orbital energies [55,56].

Thermal conductivity can be decomposed into phonon ($k_{L}$) and electron contributions ($k_{e}$):

(5) $$k=k_{L}+k_{e}.$$

Phonon contribution is called lattice thermal conductivity, which is due to energy transport by phonons, shown as follows:

(6) $$k_{L}=\frac{1}{3}cvl,$$

where c, v, and l are the heat capacity, sound velocity, and phonon mean free path, respectively [57,58]. Electron contribution is called electronic thermal conductivity, which is proportional to the electrical conductivity times the absolute temperature according to the Wiedemann‒Franz law [59]:

(7) $$k_{e}=L\sigma T,$$

where L is the Lorenz number. When materials have a low electrical conductivity, the lattice thermal conductivity becomes dorminate and the electronic thermal conductivity makes fewer or negligible contributions when the electrical conductivity <1 S/cm. The electronic thermal conductivity can be comparable to or even larger than the lattice thermal conductivity when the materials have an electrical conductivity ~100 S/cm.

The ideal TE materials should have a high Seebeck coefficient, high electrical conductivity, and low thermal conductivity [60]. However, from the above analysis, we see that these parameters are not independent of each other: the Seebeck coefficient is typically inversely proportional to the electrical conductivity; the thermal conductivity is proportional to the electrical conductivity [61]. The development of high TE performance materials is generally a process of decoupling the three key parameters. Theoretical calculations indicate that making nanostructures or composites might be a solution to pursue high TE performance materials.

## 3. TE Properties of CNTs

### 3.1. TE Properties of N-Type and P-Type CNTs

The special one-dimensional (1D) structure of CNTs has attracted the attention of researchers who are pursuing high-performance TE materials since their discovery in 1991 [62]. Theoretical results from Hick et al. showed that 1D conductors or quantum wires might have high TE performance, much greater than that of both bulk materials and two-dimensional (2D) materials, in 1993 [63]. A one-band model was proposed to discuss the relationship between the structures and *ZT* values. The *ZT* values can be effectively improved by increasing the mobility along the long direction of the nanostructures or decreasing the diameter of the nanostructure for reduced thermal conductivity. With Bi_2_Te_3_ as an example, the calculated maximum *ZT* value for 1D nanomaterials is 14, which is much higher than that of the 2D (*ZT* = 2.5) and 3D (*ZT* = 0.5) materials in theory [63,64].

It is well known that CNT can exhibit both *p*-type and *n*-type TE properties by varying the different kinds of dopants. However, CNT is typically considered as a *p*-type material, in which holes are the main charge carriers [65,66,67]. However, in 2000, Bradley et al. reported that CNTs could show a negative Seebeck coefficient in a vacuum environment, indicating that the intrinsic CNT should be an *n*-type material. The reason for the *p*-type behavior of CNTs is attributed to oxygen doping. It was also demonstrated by Collins and Kong et al. that the properties of CNTs, including local density of electronic states, electrical conductivity, and Seebeck coefficient, are very sensitive to the presence of oxygen [68,69]. In a vacuum environment, the oxygen would be depleted, resulting in the intrinsic value of CNTs. To further analyze the effect of oxygen concentration on the TE performance of CNTs, Bradley et al. proposed a model to estimate the Seebeck coefficient from the density of states (DOS) of an oxygen-doped tube [68]. The equation for the semiconducting CNTs was defined as follows after a series of mathematic conversion:

(8) $$S=-\frac{\pi^{2}}{3}\frac{k_{B}}{q}k_{B}T\alpha \frac{\Delta -\delta }{2\Delta \left|\delta \right|}\mathrm{sgn}\left(D\prime_{s}\right),$$

where Δ is the bandwidth, α is the ratio of the conductivity of the semiconducting tubes to the total conductivity of tube mats, δ is a factor related to the chemical potential and bandwidth, and $D\prime_{s}$ is the derivative of the DOS for the semiconducting tube. This model can be used to explain the effect of oxygen on nanotubes, which provides a consistent explanation for the Seebeck coefficient of CNTs. These results have increased interest in exploring *p*-type and *n*-type CNTs.

#### 3.1.1. *P*-Type CNTs

*P*-type CNTs are typically made by oxidative dopants such as oxygen [70], acids [70], or chemical oxidants, e.g., tetracyanoquinodimethane (TCNQ) and its derivatives [71]. Table 1 summarizes the thermoelectric properties reported for *p*-type CNT samples. Ryu et al. reported that *p*-type CNTs can be synthesized by a variety of methods such as chemical vapor deposition, arc discharge, high-pressure carbon monoxide, etc. A homogeneous and stable CNT dispersion can be obtained by selecting solvents such as chlorosulfonic acid (CSA), *N*-methyl-2-pyrrolidone (NMP), or deionized water with sodium dodecylbenzenesulfonate (SDBS) [70].

**Figure 2:**
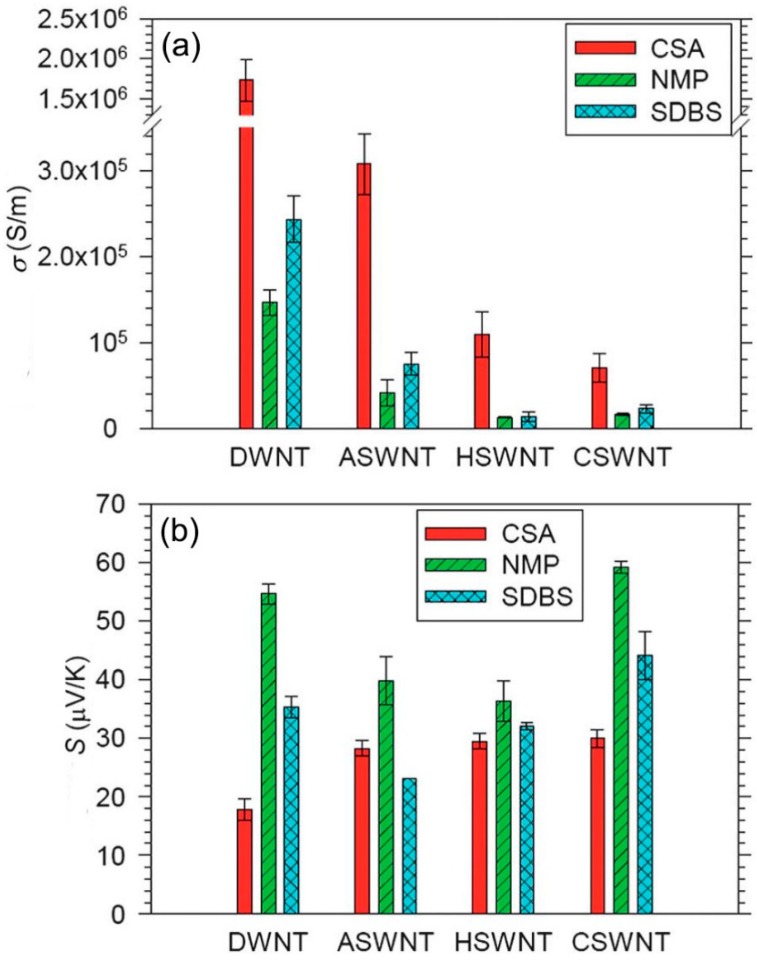
(**a**) The electrical conductivity of double-wall CNT (DWNTs), three types of single-wall CNTs made of different methods, CSWNT (chemical vapor deposition method), HSWNT (high-pressure carbon monoxide method), ASWNT (arc charge method). (**b**) The Seebeck coefficient of DWNT, CSNT, HSWNT, and ASWNT. Reproduced with permission from Ref. [70]. Copyright © Royal Society of Chemistry, 2012.

As shown in Figure 2, based on the ambient environment, all the CNTs showed positive Seebeck coefficients in the range of 20–60 μV/K, which can be explained by hole transport in the valence band [72]. This means that all of the nanotubes were doped by oxygen and showed *p*-type properties. The electrical conductivities were in the order of CSA > SDBS > NMP, while the Seebeck coefficient was in the order of CSA < SDBS < NMP. The results could be well explained with Equation (3). High doping levels moved $E_{F}$ into the conduction band and led to the number of electronic states above and below $E_{F}$ being more equivalent, subsequently resulting in an increased σ and reduced S. The polarity of the three solvents should be in the order of CSA > SDBS > NMP. In NMP, the doping level of CNTs was the lowest, thus the CNTs had the highest S and lowest σ among these three solutions. In the solution of SDBS, the doping level of CNTs was higher than that in NMP since there were more hydrogen ions from water. In CSA, the intercalation of hydrogen ions between the nanotubes and oxidation of CNTs by CSA further increased the doping level, thus increasing the σ and reducing the S [73].

To further improve the *p*-type Seebeck coefficient of CNTs, an Ar-plasma treatment method has been reported. The results show that CNTs can achieve a high Seebeck coefficient over 300 μV/K at 670 K after 20 s of Ar-plasma treatment [74]. Although the electrical conductivity decreased from 3500–4800 to 330–990 S/m, the power factor increased to over 120 μV/K. The *ZT* value could reach about 0.4 because the thermal conductivity of the CNT film was measured to be as low as 0.28–0.34 W/m⋅K after 20 s Ar-plasma treatment [74]. Furthermore, the decrease in electrical conductivity and the increase in the Seebeck coefficient are mainly attributed to the large number of defects created by Ar plasma.

#### 3.1.2. *N*-Type CNTs

Table 2 summarizes the TE properties reported for *n*-type CNT samples. It is noted that *n*-type CNTs are much more challenging to obtain compared with the *p*-type CNT, since the oxygen in air makes most CNTs have *p*-type properties. [75] A common strategy to convert *p*-type CNTs into *n*-type is to use reduction chemicals such as NaBH_4_ and hydrazine [76]. However, the reduced CNTs are not stable in air because they will be oxidized again and converted to *p-type* soon. Therefore, chemicals with electron-donating groups, such as poly(ethyleneimine) (PEI) [77,78,79], reduced viologen [33,80], rylene diimide [81], etc. are generally used as *n*-type dopants and have been demonstrated to have relatively stable *n*-type doping effects.

**Figure 3:**
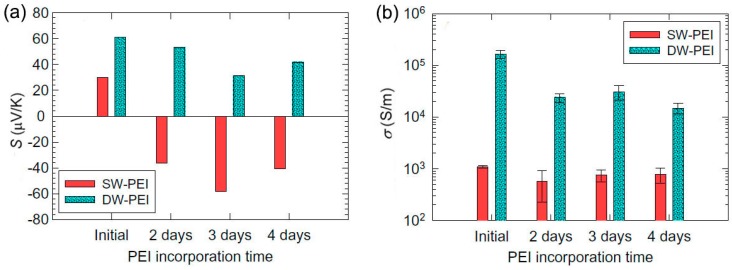
Seebeck coefficient (**a**) and electrical conductivity (**b**) of the SWCNT (SW) and DWCNT (DW) films before and after PEI incorporation for two, three, and four days. PEI can convert SWCNT rather than DWCNT from *p*-type to *n*-type. Reproduced with permission from Ref. [75]. Copyright © Elsevier, 2011.

PEI represents a type of insulating *n*-type dopants. The TE properties of PEI-doped CNTs have been tested by Ryu et al., who showed a Seebeck coefficient of −58 μV/K with a good electrical conductivity of ~10^5^ S/m (Figure 3) [75]. PEI can provide electrons to nanotubes, which moves the Fermi level of nanotubes toward the lowest unoccupied molecular orbital (LUMO), resulting in *n*-type doping. DWCNTs cannot be converted from *p*-type to *n*-type, which is attributed to the large hole concentration in DWCNTs. After a long incorporation time of 2‒3 days, the Seebeck coefficient value of DWCNTs decreased, which might be due to the fact that some holes were canceled out by electrons [75].

**Figure 4:**
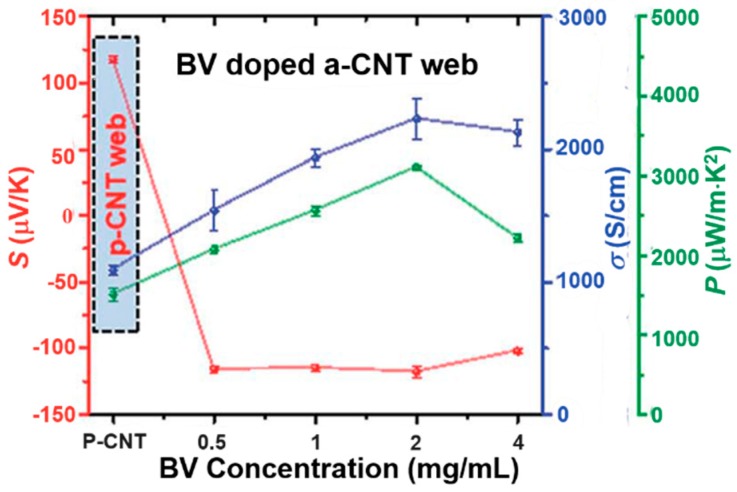
Seebeck coefficient, electrical conductivity and power factor of BV-doped CNT. The pristine CNT after annealing shows a Seebeck coefficient of 117.7 μV/K. After being doped with 0.5 mg/mL BV, the Seebeck coefficient changed to −116 μV/K. Further increasing the concentration of BV has little effect on the Seebeck coefficient. Reproduced with permission from Ref. [82]. Copyright © Royal Society of Chemistry, 2017.

Viologen can convert *p*-type CNTs to *n*-type through an electrochemical redox reaction. Kim and Biswas et al. reported that an environmentally stable *n*-type CNT could be made when using reduction-controlled viologen as a dopant [80,82,83]. The positively charged viologen (V^2+^) was reduced to neutral (V^0^), which was dissolved in toluene. The solution was then dropped onto a CNTs film, which then showed *n*-type transistor properties [80]. Half of the CNT films treated with viologen showed good rectification properties [83]. The TE properties of viologen-doped CNTs were tested by An et al. [82], who showed a high Seebeck coefficient of −116 μV/K with a high electrical conductivity of 1534 S/cm, leading to a high power factor of 3103 μW/m K^2^ as shown in Figure 4. The obtained CNT films showed a relatively low in-plane thermal conductivity of ~5 W/m K, resulting in a high *ZT* value of 0.2.

**Figure 5:**
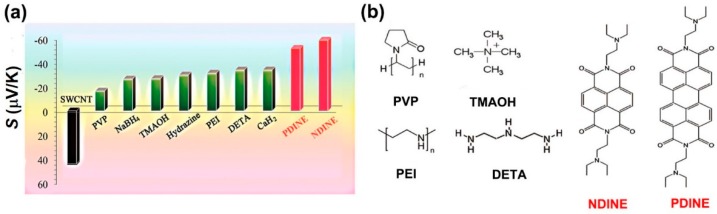
(**a**) Seebeck coefficients of the films of the pristine SWCNT and the SWCNTs treated by the dopants. (**b**) Molecular structures of the related *n*-type dopants. Reproduced with permission from Ref. [81]. Copyright © American Chemical Society, 2017.

Some of the eylene diimide derivatives are conductive *n*-type dopants. Wu et al. reported that perylene diimide (PDINE) and naphthalene diimide (NDINE)-doped CNTs have good Seebeck coefficients of −52.4 and −60.2 μV/K, respectively. These values are larger than that of the PEI treated by reduction chemicals and insulating electron-donating compounds, as shown in Figure 5. In addition, PDINE/CNTs and NDINE/CNTs showed good stability in air. After 100 h exposing in air, the hybrids retain >80% of electrical conductivity and >70% of Seebeck coefficient. The hybrids also showed good stability while being heated. At 200 °C, PDINE/CNTs and NDINE/CNTs can maintain 97.8% and 96.6% of weight, respectively, while PEI/CNTs can only maintain 83.5% of weight. The high stability of PDINE/CNTs and NDINE/CNTs might be attributed to the strong π‒π interactions between the molecules and CNTs. As we can see that most of the *n*-type dopants contains nitrogen atoms, this might be the key to designing good *n*-type dopants.

#### 3.1.3. CNTs-Based TE Devices

**Figure 6:**
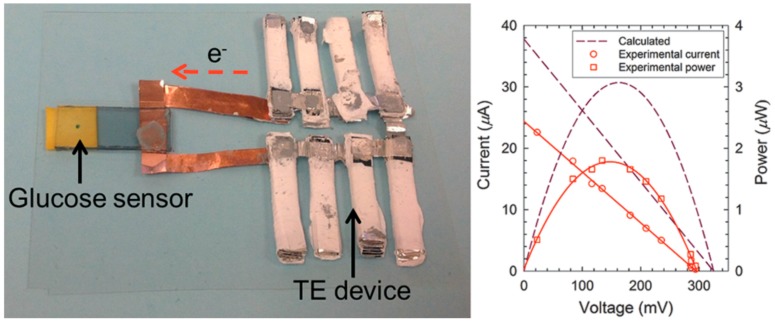
Prototype of glucose detection sensor integrated with a TE device. The glucose solution was dropped on the testing plate. When power (150 mV) was supplied by the TE device, the Prussian blue disappeared after 180 s due to the change into Prussian white. Reproduced with permission from Ref. [85]. Copyright © American Chemical Society, 2014.

CNTs have good mechanical properties that make them suitable for use in TE devices. Yu et al. reported that CNTs-based leg structure modules can produce ~6 mV TE voltage with three *p*‒*n* couples and generate ~25 nW power upon the application of a temperature gradient of ~22 K [84]. *P*-type modules were made of CNT films with SDBS as a dispersant and *n*-type modules were made of CNT films with PEI as a dispersant and dopant. The Seebeck coefficients of *p*-type and *n*-type CNTs were measured as 81 and −80 μV/K, respectively. Further work with salable *p*‒*n* couple modules may enhance the power output of this sample construction method. The application of the TE device was studied by the sample group. A glucose sensor was made with a TE device as the power source, as shown in Figure 6. The TE device contains 72 *p*-type and 72 *n-*type CNT modules that can produce 150 mV at a temperature gradient of 32 K. The power output was 1.8 μW, which is practically viable for operating sensor units equipped with wireless communication and power management circuits.

**Figure 7:**
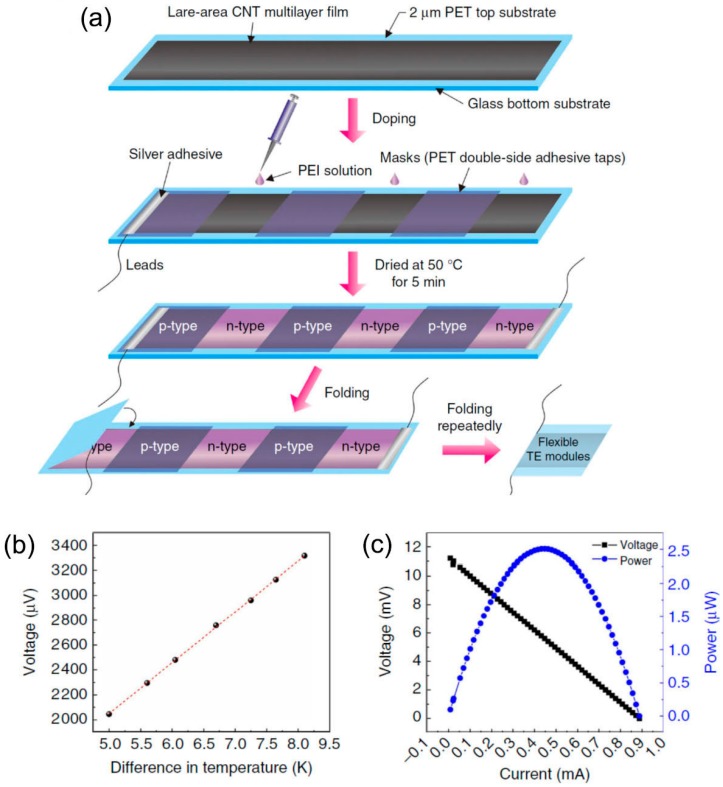
(**a**) Preparation of compact-designed TE modules. A novel configuration, compact and efficient flexible TE module based on large-area continuously synthesized CNT films and localized doping technology. (**b**) The output voltage of the module depends on the different temperature gradient. (**c**) The relationship between output voltage, output power, and output current at the hot-side temperature of 330 K and temperature difference of 27.5 K. Reproduced with permission from Ref. [86]. Copyright © Springer Nature, 2017.

**Figure 8:**
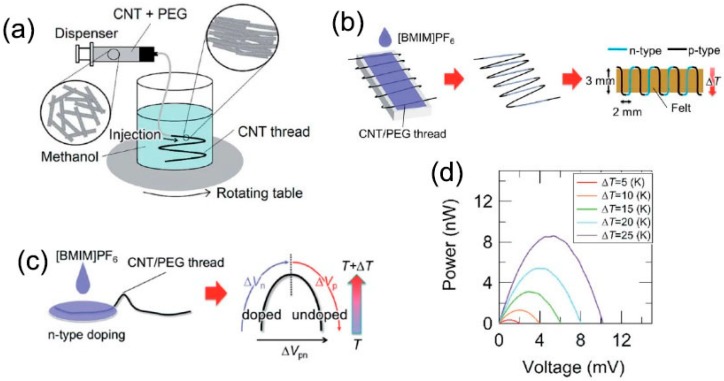
(**a**) Schematic drawing of the wet-spinning method whereby the CNT dispersion is injected into a coagulant in a rotating vessel. (**b**) Schematic drawing of the fabrication process of a “TE fabric.” (**c**) Schematic illustration showing partial *n-*type doping and TE measurement of the doped part (Δ*V*_n_), the undoped part (Δ*V*_p_), and the sum of them (Δ*V*_pn_). (**d**) Output characteristics of a prototype TE fabric. Reproduced with permission from Ref. [87]. Copyright © Royal Society of Chemistry, 2013.

Compact-design, flexible TE modules were reported in 2017 by Zhou et al. [86], showing good stability for over three months (the variations in electrical conductivity and Seebeck coefficient were less than 5%) in air without encapsulation. The modules were fabricated on continuous CNT networks as shown in Figure 7. PEI solution was drop-casted into several CNT ribbons. The effect of the concentration of PEI solution on the TE performance of CNTs was investigated systematically. When the PEI concentration is 1 wt %, the optimized *n*-type films show an ultrahigh power factor of ~1500 μW/m K^2^. This method simplified the module preparation process, which indicates that the roll-to-roll technique can be used for large, scalable CNT-based modules. Additionally, Mitsuhiro et al. [87] proposed the direct injection pyrolytic synthesis (DIPS) method with the reinforcing agent of PEG to prepare a *p*-type CNT/PEG thread, as shown in Figure 8 [88]. The Seebeck coefficient of the *p*-type CNT/PEG thread is 47.8 μV/K, which is slightly lower than mentioned above. Furthermore, the *n*-type dopant 1-butyl-3-methylimidazolium hexafluorophosphate ([BMIM]PF6) can be used to obtain *n*-type CNT/PEG thread with a Seebeck coefficient stable at −49.1 μV/K. Finally, a novel prototype TE fabric is fabricated by using single CNT/PEG thread, p/n-striped doping, and easy fabrication processes of π-type cells. The output performance of the prototype TE fabric is investigated. This novel method can not only fabricate wearable flexible *p*/*n*-type TE devices by a single CNT-based thread, but also reduce the interface contact resistance to effectively improve the output performance.

### 3.2. TE Performance of Semiconducting CNTs

**Figure 9:**
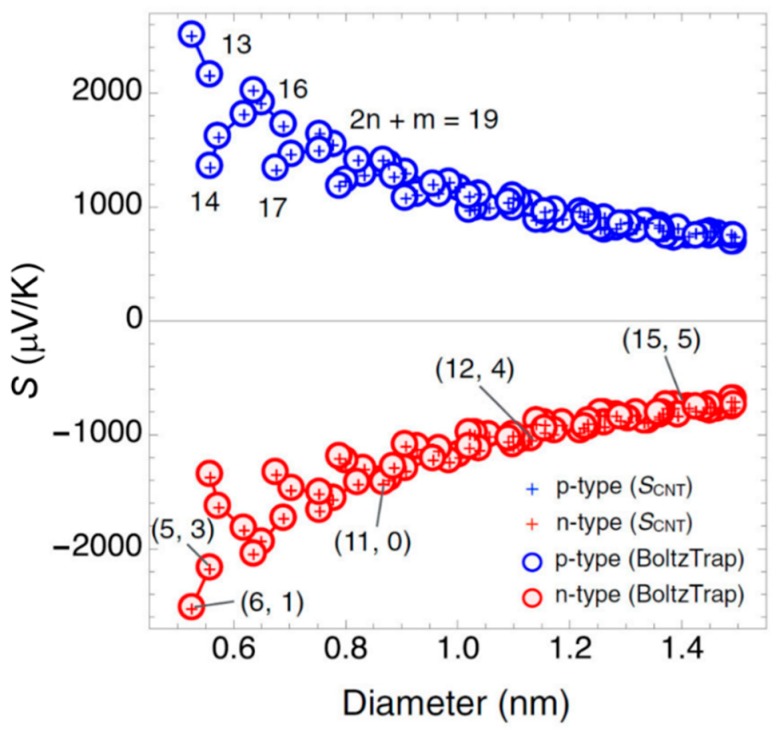
Seebeck coefficient of semiconducting CNTs within the diameter range 0.5–1.5 nm. The temperature is constant at 300 K. Reproduced with permission from Ref. [93]. Copyright © American Physical Society, 2015.

One-dimensional structure materials could have a high *ZT* value, as mentioned in Section 3.1, which can be attributed to the quantum confinement effect and can create sharp features in the DOS [63,89]. This reveals that CNTs should have good TE performance. However, the Seebeck coefficient of CNTs in experiments is typically in the range of 20–100 μV/K, which is lower than that of inorganic materials [90]. A theoretical study of the relationship between Seebeck coefficient and diameter has been reported by Hung et al. [91]. A simple formula can be used to calculate the Seebeck coefficient of semiconducting CNTs, which is derived from their band gap:

(9) $$S=\frac{k_{B}}{q}\left(\frac{\mu }{k_{B}T}-\frac{E_{g}}{2k_{B}T}-\frac{3}{2}+\frac{\frac{E_{g}}{k_{B}T}+3}{q^{2\mu /k_{B}T}+1}\right),$$

where $E_{g}$ is the CNT band gap that is obtained from the extended-tight binding results reported in a previous paper [92]. As shown in Figure 9, the Seebeck coefficient values decrease with the increase of CNT diameter. Furthermore, it is proven that the Seebeck coefficient of an individual semiconducting CNTs can reach up to 2000 μV/K at room temperature for diameters less than 0.6 nm, such as the (5, 3) and (6, 1) semiconducting CNTs pointed out in Figure 9. The low experimental values are suggested to be attributed to the complex of geometrical and electronic structures of CNTs [67,93], because the potential TE properties might have been lost due to the interactions between different tubes [94]. Therefore, the separation of CNTs is essential for obtaining high-purity CNTs with even single charity. 

**Figure 10:**
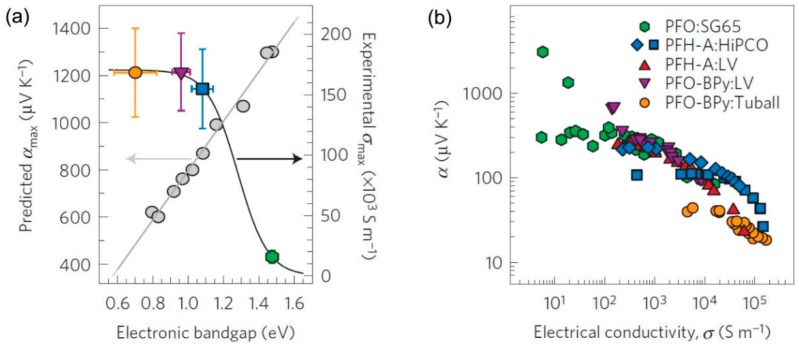
(**a**) Dependence of the theoretically predicted peak Seebeck coefficient (grey symbols and grey line) and maximum experimentally measured electrical conductivity (colored symbols and black line) on the semiconducting CNT electronic bandgap. The vertical error bars are derived from the standard deviation of the measured sheet resistances and film thicknesses. The horizontal error bars denote the standard deviation of the electronic bandgap derived from the semiconducting CNT diameter distribution. (**b**) Experimentally measured Seebeck coefficient as a function of electrical conductivity Reproduced with permission from Ref. [101]. Copyright © Springer Nature, 2016.

Theoretical results have attracted interest to exploring high TE performance materials with high-purity semiconducting CNTs [95,96]. Recently, with the development of several sorting strategies [97,98,99] and semiconducting CNTs synthesis methods [100], the production of selected diameter or electronic type CNTs has been realized. The TE properties of high-purity semiconducting CNTs have been tested. Avery et al. [101] reported that semiconducting SWCNTs with carefully controlled chirality distribution and carrier density can have a large TE power factor of 340 μW/m K^2^. As shown in Figure 10a, the predicted Seebeck coefficients are in the range of 400–1200 μV/K, which varies with the electronic band gap (diameter). In the experiments shown in Figure 10b, the measured Seebeck coefficients are generally in the range of 20–300 μV/K. Only a few samples can exhibit a huge Seebeck coefficient over 1000 μV/K. The calculated *ZT* values are in the range of 0.01–0.05. The relatively low experimental values are attributed to the existence of polymers and the semiconducting CNT diameter distribution. Although the results are not very impressive, they indicate a promising way to further improve the TE performance of CNT-based TE materials by using semiconducting CNT species. For instance, large-diameter semiconducting CNTs could enhance the electrical conductivity, while small-diameter semiconducting CNTs could raise the Seebeck coefficient.

**Figure 11:**
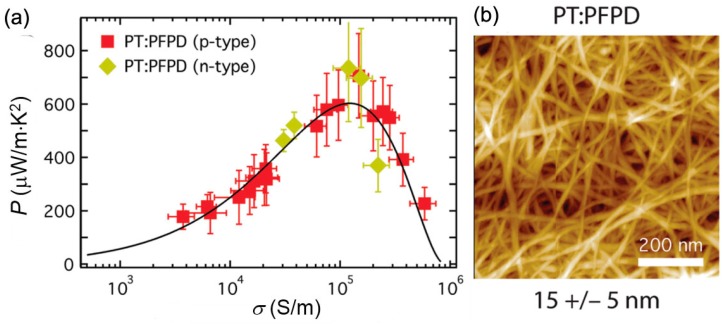
(**a**) *n*- and *p*-type power factor for semiconducting CNT networks. (**b**) Atomic force microscope images of semiconducting CNT networks prepared from PT: PFPD dispersions. PT: plasma torch; PFPD: poly[(9,9-di-*n*-dodecyl-2,7-fluorendiyl-dimethine)-(1,4-phenylene-dinitrilomethine)]. Reproduced with permission from Ref. [102]. Copyright © Royal Society of Chemistry, 2017.

To further improve the TE performance of semiconducting CNTs, a method has been developed by MacLeod et al. to remove the polymer species [102]. A high power factor of 700 μW/m K^2^ at 298 K for the highly enriched CNT thin films, containing 100% semiconducting CNTs, is realized. The semiconducting CNTs are sorted by PFPD and then doped with *n*- and *p*-type molecular dopants. The PFPD polymer can be partially washed off. As shown in Figure 11, a densely packed CNT network is obtained. A peak power factor for both *n*- and *p*-type CNTs can be obtained while tuning the doping level. The thermal conductivity of the networks is measured to be about 2 W/m K, leading to a peak *ZT* of 0.12 for semiconducting CNTs with a diameter in the range of 1.0 nm.

Besides the advantages of light weight, flexibility, and solution process capability, CNTs can be made into *n*- and *p-*type materials, leading to the potential application of full CNTs TE devices. In addition, they are much more resistant to thermal degradation than polymers, and thus have a broader range of operating temperatures, up to 200 °C. The findings of high TE properties of semiconducting CNTs provide a potential way to further improve the TE performance.

## 4. CNT‒Polymer Composites

### 4.1. In Series and Parallel Models for Composites

Making hybrids is an alternative strategy to obtain materials with desirable properties by combining the advantages of two or more components [103,104]. In a binary TE composite system, a series or parallel model is typically used to evaluate the Seebeck coefficient, electrical conductivity, and thermal conductivity of the composites [105,106,107,108]. Assuming that Material A has $S_{1}$, $\sigma_{1}$ $k_{1}$ and Material B has $S_{2}$, $\sigma_{2}$ $k_{2}$, in a series model (Equations (10)–(12)):

(10) $$\;S=\frac{S_{1}*\left(\frac{1}{k_{1}}\right)*x}{\frac{1}{k_{1}}*x+\frac{1}{k_{2}}*\left(1-x\right)}+\frac{S_{2}*\left(\frac{1}{k_{2}}\right)*\left(1-x\right)}{\frac{1}{k_{1}}*x+\frac{1}{k_{2}}*\left(1-x\right)}\;$$

(11) $$\sigma =\frac{\sigma_{1}*\sigma_{2}}{x*\sigma_{2}+\left(1-x\right)*\sigma_{1}}\;$$

(12) $$k=\left(1-x\right)k_{1}+xk_{2}.$$

In a parallel model (Equations (13)–(15)),

(13) $$S=\frac{S_{1}*\sigma_{1}*x+S_{2}*\sigma_{2}*\left(1-x\right)}{\sigma_{1}*x+\sigma_{2}*\left(1-x\right)}\;$$

(14) $$\sigma =\sigma_{1}*x+\sigma_{2}*\left(1-x\right)\;$$

(15) $$\frac{1}{k}=\frac{1-x}{k_{1}}+\frac{x}{k_{2}},$$

where x is the volume ratio for Material A (Material A is the discrete phase) [109]. The theoretical value calculated from the above equations typically does not match the experimental results. As we can see, these models are pretty simple and do not consider the sample preparation conditions, quantum effects, interfaces between the two materials, etc. [108]. These models can only provide us with a rough qualitative analysis of the TE properties of the composite.

### 4.2. TE Properties of CNT‒Polymer Composite

**Figure 12:**
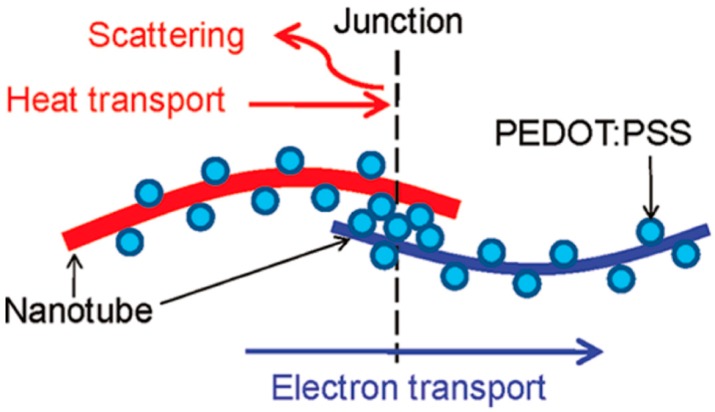
PEDOT:PSS particles are coated on CNTs, making CNT‒PEDOT:PSS‒CNT junctions in the composites. It is hypothesized that the presence of the junction can deter heat transport without affecting electron transport much. Reproduced with permission from Ref. [102]. Copyright © American Chemical Society, 2011.

With the idea of integrating the bilateral advantages of each component, CNT‒polymer composites have been proposed [110,111,112]. Table 3 summarizes the TE properties reported for CNT‒polymer composite samples. In the beginning, polymer is used as a poor thermal conductor in the composites since the intrinsic thermal conductivity of the polymers is low [113]. A segregated network CNT‒polymer structure with an electrical conductivity of 4800 S/m, thermal conductivity of 0.34 W/m K, and *ZT* value of 0.006 at room temperature has been reported [114]. Since then, with the fast development of conducting polymers, poly(3,4-ethylenedioxythiophene): poly(styrenesulfonate) (PEDOT:PSS) has become one of the most widely used polymers [115]. A well-known junction structure has been proposed as shown in Figure 12, which is believed to give rise to exceptional TE transport properties. Specifically, a series of connected electron channels formed at the interfaces. Because of the excellent electronic transmission properties of CNT, the electrical conductivity of the composite film increased significantly to ~40,000 S/m. In addition, due to the mismatch of the molecular vibrational spectra between CNT and PEDOT:PSS, the propagation of phonons at the nodes is impeded, thus preventing the increase of thermal conductivity and the decrease of the Seebeck coefficient. Finally, the optimized *ZT* value reached 0.02 at room temperature [116]. Furthermore, the TE performance is found to increase with the increase in the CNT ratio. When the concentration of CNTs reaches 60 wt % in the CNT‒polymer composite, an electrical conductivity of 1.3 × 10^5^ S/m is obtained with a Seebeck coefficient value of 41 μV/K [117]. The out-of-plane thermal conductivity is measured to be 0.2–0.4 W/m K. However, the *ZT* value is not provided in the paper since the electrical properties are all in the in-plane direction. It should be mentioned that morphological factors such as the distribution of the CNT in the polymer matrix and the roughness of the surface of the composites, also play an important role in determining the ultimate TE power factor.

**Figure 13:**
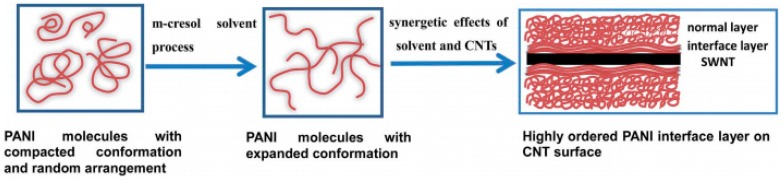
Schematic representation of the formation of an ordered PANI interface layer induced by the synergistic effects of the solvent process and the π‒π interactions conjugation between CNTs and PANI. Reproduced with permission from Ref. [119]. Copyright © Royal Society of Chemistry, 2014.

CNTs can also be used as a template to align the chains of conducting polymers [118]. Yao et al. have reported that CNT‒polyaniline (PANI) exhibits a high electrical conductivity of 76,900 S/m, a high Seebeck coefficient of 65 μV/K, and a low thermal conductivity of 0.43 W/m K, leading to a *ZT* value of 0.12 at room temperature [119]. In the CNT‒PANI composites, it has been demonstrated that the PANI chain will pack densely on CNT surfaces due to the strong π‒π interactions between CNTs and PANI, as shown in Figure 13. The good crystallinity of polymers in the composite is the main reason for the high TE properties of the CNT‒PANI composite.

**Figure 14:**
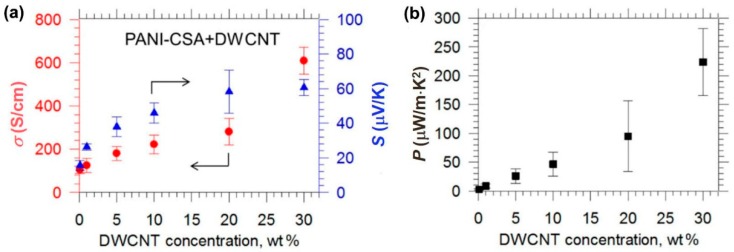
(**a**) Electrical conductivity and Seebeck coefficient of CNT‒PANI composites as a function of CNT concentration. (**b**) The corresponding power factors of the composite. DWCNT: double wall carbon nanotube. PANI‒CSA: polyaniline‒camphorsulfonic acid. Reproduced with permission from Ref. [121]. Copyright © American Chemical Society, 2015.

Simultaneous enhancement of electrical conductivity and Seebeck coefficient has been observed in the CNT‒polymer composites, which is unusual and does not follow the Wiedemann‒Franz law [120]. Typically, an increase in electrical conductivity accompanies a decrease in the Seebeck coefficient, and vice versa. Hong et al. reported that the electrical conductivity and Seebeck coefficient can be simultaneously increased [121]. As shown in Figure 14, the CNT‒PANI composite has a high electrical conductivity of 61,000 S/m and a high Seebeck coefficient of 61 μV/K, leading to a high power factor of 220 μW/m K^2^, which is the highest among previously reported PANI-based TE materials. The main reason for the simultaneously enhancement of electrical conductivity and Seebeck coefficient is due to the improvement of carrier mobility that raises the electrical conductivity and the reduction of carrier concentration that enlarges the Seebeck coefficient. The improvement in carrier mobility is attributed to the band alignment, which attracts hole carriers to CNTs whose mobility is much higher than that of the polymer. In addition, the energy barrier at the interface of CNT and PANI may play an important role [122,123,124,125,126]. Theoretical study suggests that the power factor can be remarkably improved by imposing an energy barrier at the interface of conjugated carbon structures [118,127,128].

**Figure 15:**
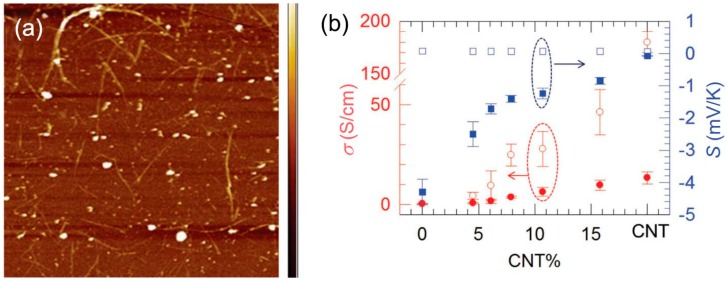
(**a**) Atomic force microscope image of non-percolated CNT network on a glass substrate. (**b**) Characterization of electrical properties of the composite before and after tetrakis(dimethylamino)ethylene (TDAE) reduction. Empty dots are for samples before TDAE treatment. Solid dots are for samples after TDAE treatment. Reproduced with permission from Ref. [121]. Copyright © John Wiley and Sons, 2015.

A novel non-percolated structure has been prepared to further increase the TE properties of CNT‒polymer composites as shown in Figure 15, in which CNTs serves as a high carrier mobility quantum well And the CNT‒polymer‒CNT junctions are formed, leading to a significantly reduced thermal conductivity [38]. The non-percolated CNT networks are prepared on a glass substrate by the spray method. Then the monomer is spin-coated on the CNT sample, followed by in situ polymerization. Finally, the sample is reduced by TDAE to have a high Seebeck coefficient. The maximum power factor obtained by this method is up to 1050 μW/m K^2^, with a thermal conductivity of ~0.65 W/m K. The obtained *ZT* is about 0.5, which is considered the highest *ZT* value among organic TE materials [129].

## 5. Conclusions

CNTs are one-dimensional conductors that might have higher TE performance than bulk and 2D materials in theory. In the past decade, progress has been made in developing high TE performance organic materials with CNTs. In this review, CNT-based and CNT‒polymer-based TE materials have been discussed in detail, including the intrinsic TE properties of CNTs, strategies for tuning the TE properties of CNTs, and the development of CNT‒polymer composites. CNT is a very promising TE material. The ease of making both *n*-type and *p*-type TE modules with CNTs provides a potentially simple fabrication method to prepare full CNT devices, which have a broader operating temperature (above 200 °C) than traditional polymers. In addition, there is still room to improve the properties of CNTs since a high Seebeck coefficient—up to 2000 μV/K in theory—is still unachievable in experiments. A promising result has been reported recently with a *ZT* value of 0.5 for CNT‒polymer composites. Impressive results have been achieved in the past decade, which indicates the promising future of CNT‒ and CNT‒polymer composite-based TE generators as power sources for sensors, sophisticated medical devices, and in the security and environmental sectors.

## Figures and Tables

**Table 1:** Thermoelectric properties of the *p*-type CNT samples at room temperature.

Materials	Electrical Conductivity [S/cm]	Seebeck Coefficient [μV/K]	Power Factor [μW/m K^2^]	Ref.
DWNT-CSA	~1.7 × 10^4^	~18	550.80	[70]
DWNT-NMP	~1500	~55	453.75	[70]
DWNT-SDBS	~2400	~36	311.04	[70]
ASWNT-CSA	~3100	~28	243.04	[70]
ASWNT-NMP	~500	~40	80	[70]
ASWNT-SDBS	~700	~24	40.32	[70]

**Table 2:** Thermoelectric properties of the *n*-type CNT samples at room temperature.

Materials	Electrical Conductivity [S cm^−1^]	Seebeck Coefficient [μV K^−1^]	Power Factor [μW m^−1^ K^−2^]	Ref.
SW-PEI	10	−58	3.4	[75]
DWNT-PEI/graphene-PVP	297	−80	190	[76]
SWCNT-PDINE	500	−47.3	112 ± 8	[81]
SWCNT-NDINE	446	−55	135 ± 14	[81]

**Table 3:** TE properties of the CNT‒polymer-based composite at room temperature.

Materials	Electrical Conductivity [S cm^−1^]	Seebeck Coefficient [μV K^−1^]	Power Factor [μW m^−1^ K^−2]^	Thermal Conductivity [W m^−1^ K^−1^]	ZT	Ref.
Hydrophilic pyridinium salt polymer/SWCNTs	159	63.3	46.4	-	-	[110]
Poly-Schiff base/SWCNT	411.8 ± 18.7	43.4 ± 0.7	77.7 ± 5.8	-	-	[111]
PEDOT:SWCNT	1444.5 ± 86.9	53.9 ± 2.9	253.7 ± 10.4	-	-	[112]
Segregated-network CNT−polymer	48	38	6.9	0.34	0.006	[114]
CNT‒PEDOT:PSS	400	18.3	13.4	0.2–0.4	0.02	[116]
SWNT/PANI	769	65	176	0.43	0.12	[119]
CNT/PANI‒CSA	610	61	220	-	-	[121]
